# Drivers of Inequalities among Families Involved with Child Welfare Services: A General Overview

**DOI:** 10.3390/ijerph19137881

**Published:** 2022-06-27

**Authors:** Ylva B. Almquist, Viviane S. Straatmann

**Affiliations:** Department of Public Health Sciences, Stockholm University, 106 91 Stockholm, Sweden

When parents are unable to raise their children or to meet the minimum acceptable standards for their care, this can lead to involvement with child welfare services. In some cases, children are separated from their families and taken into out-of-home care (OHC). For good reason, prior research has primarily focused on the lifelong development of these children, and there is now extensive evidence showing that the experience of placement in OHC is predictive of worse outcomes in almost every dimension of adult life [1,2,3,4,5,6]. It is nonetheless likely that the treatment of OHC as a risk factor at the level of the individual child greatly underestimates the scope of the issue from a public health perspective. Here, we argue that gaining more knowledge about the familial circumstances under which the child welfare services enter—and, in many cases, later on exit—the scene would not only facilitate the understanding of why experiences of OHC tend to leave such long-lasting marks on children, but also how they reflect and contribute to inequalities at the population level.

Primary reasons for children being placed in OHC include problems that can be said to broadly reflect poor mental health (e.g., affective disorders, substance misuse, and self-harm) among the biological parents [7]. Importantly, however, the families that come to the attention of the child welfare services also diverge in other systematic ways from the general population. For example, the biological parents are much more likely to live under disadvantaged socioeconomic conditions (as often indicated by single parenthood, belonging to minority groups, poverty, low education, and poor labor market attachment) [7,8,9]. Past inquiries have commonly studied these types of socioeconomic disadvantage without discerning the weight and contribution of each factor [10]. The extent to which they actually drive the selection of children into OHC is thus still uncertain. There is furthermore a complex interplay between socioeconomic disadvantages and mental health problems; for example, parental substance use accompanied by poverty is more likely to lead to involvement with child welfare services than substance use in a position of affluence [8]. Disentangling how these processes at the familial level may—or may not—lead to the placement of a child in OHC would make important contributions to our understanding of the drivers of inequalities among families involved with child welfare services. This could in turn help us to find actionable entry points and assist the development of effective policies to reduce such inequalities.

After a child has been placed in OHC, what happens to the parents? Hitherto, studies on consequences for biological parents have been limited, particularly with regard to research based on quantitative designs. There is thus a lack of knowledge about whether the placement is actually followed by improvement or deterioration among the parents. On the one hand, in the face of other troublesome circumstances, not having to care for the child could relieve stress [11]. Parents might also make strong efforts to improve their situation in order to increase the chances of family reunification. On the other hand, recent studies have shown that having a child taken into OHC can have severe negative consequences, including (worsening) mental health problems (e.g., grief, suicidality, depression, anxiety, post-traumatic stress, and substance use) and premature mortality [12,13,14]. Other research has pointed to elevated social and economic disadvantages, including increased stigma, housing instability, loss of income, and weakened social support [14,15,16]. Though fathers have often been overlooked in this kind of research, there are studies to suggest that their health seems to be less affected by having a child placed in OHC as compared to the health of the mothers [1,17,18]. Nonetheless, there is some indication of a clearer ‘downward spiral’ effect when it comes to socioeconomic conditions among fathers: having a child placed in OHC appears to further aggravate the father’s conditions, leading to a longer duration of a child’s OHC placement [19]. In some countries—such as Sweden—OHC is generally not a permanent intervention: most placements are short-term with the more or less explicit goal of reuniting children to their biological parents [20,21]. The viability of this ambition remains unclear, particularly when considering the shortage of supportive programs targeting the biological parents’ health and wellbeing [21]. Both parents and social workers have also expressed a need for more practical and financial aid to the family [22]. Not focusing on improving the overall conditions of families with children in OHC might be particularly problematic in cases where the placement further exacerbates the disadvantaged situation of the mother and/or the father [21,23].

This Special Issue encourages researchers to explore inequalities related to involvement with child welfare services. More specifically, we call for theoretical and empirical contributions that inquire into the familial circumstances that precede and succeed the placement of a child into OHC from an inequality perspective. The overall ambition of the Special Issue is to inform efforts into preventing the conditions of biological parents from reaching a level that requires that the child is placed into OHC, but also efforts aiming to improve the conditions of families once the placement has occurred.
